# HLA-A*0201-restricted CD8^+^ T-cell epitopes identified in dengue viruses

**DOI:** 10.1186/1743-422X-9-259

**Published:** 2012-11-05

**Authors:** Zhi-Liang Duan, Qiang Li, Zhi-Bin Wang, Ke-Dong Xia, Jiang-Long Guo, Wen-Quan Liu, Jin-Sheng Wen

**Affiliations:** 1 Department of Microbiology and Immunology, Wenzhou Medical College, Wenzhou, China; 2 Department of Clinical Laboratory, The Second Affiliated Hospital of Wenzhou Medical College, Wenzhou, China; 3 Department of Clinical Laboratory, The people’s Hospital of Ruian, Wenzhou, China

**Keywords:** Dengue virus, CD8^+^ T-cell epitope, Immunogenicity

## Abstract

**Background:**

All four dengue virus (DV) serotypes (D1V, D2V, D3V and D4V) can cause a series of disorders, ranging from mild dengue fever (DF) to severe dengue hemorrhagic fever and dengue shock syndrome (DHF/DSS). Previous studies have revealed that DV serotype-specific CD8^+^ T cells are involved in controlling DV infection. Serotype cross-reactive CD8^+^ T-cells may contribute to the immunopathogenesis of DHF/DSS. The aim of the study was to identify HLA-A*0201-binding peptides from four DV serotypes. We then examined their immunogenicity *in vivo* and cross-reactivity within heterologous peptides.

**Methods:**

D1V-derived candidate CD8^+^ T-cell epitopes were synthesized and evaluated for their affinity to the HLA-A*0201 molecule. Variant peptides representing heterologous D2V, D3V, D4V serotypes were synthesized. The immunogenicity of the high-affinity peptides were evaluated in HLA-A*0201 transgenic mice.

**Results:**

Of the seven D1V-derived candidate epitopes [D1V-NS4a_56–64_(MLLALIAVL), D1V-C_46–54_(LVMAFMAFL), D1V-NS4b_562–570_(LLATSIFKL), D1V-NS2a_169–177_(AMVLSIVSL), D1V-NS4a_140–148_(GLLFMILTV), D1V-NS2a_144–152_(QLWAALLSL) and D1V-NS4b_183–191_(LLMRTTWAL)], three peptides [D1V-NS4a_140–148_, D1V-NS2a_144–152_ and D1V-NS4b_183–191_] had a high affinity for HLA-A*0201 molecules. Moreover, their variant peptides for D2V, D3V and D4V [D2V-NS4a_140–148_(AILTVVAAT), D3V-NS4a_140-148_(GILTLAAIV), D4V-NS4a_140-148_(TILTIIGLI), D2V-NS2a_144–152_(QLAVTIMAI), D3V-NS2a_144–152_(QLWTALVSL), D4V-NS2a_143–151_(QVGTLALSL), D2V-NS4b_182–190_(LMMRTTWAL)_,_ D3V-NS4b_182–190_ (LLMRTSWAL) and D4V-NS4b_179–187_(LLMRTTWAF)] also had a high affinity for HLA-A*0201 molecules. Furthermore, CD8^+^ T cells directed to these twelve peptides were induced in HLA-A*0201 transgenic mice following immunization with these peptides. Additionally, cross-reactivity within four peptides (D1V-NS4b_183–191_, D2V-NS4b_182–190,_ D3V-NS4b_182–190_ and D4V-NS4b_179–187_) was observed.

**Conclusions:**

Two novel serotype-specific HLA-A*0201-restricted CD8^+^ T-cell epitopes (NS4a_140-148_ and NS2a_144–152_) and one cross-reactive HLA-A*0201-restricted CD8^+^ T-cell epitopes which is similar to a previously identified epitope were identified in D1V-D4V. Combining prediction algorithms and HLA transgenic mice is an effective strategy to identify HLA-restricted epitopes. Serotype-specific epitopes would be used to determine the protective role of serotype-specific CD8^+^ T cells, while cross-reactive epitopes may provide assistance in exploring the role of serotype cross-reactive CD8^+^ T cells in the immunopathogenesis of DHF/DSS.

## Background

Dengue virus (DV) is a single-stranded positive-sense RNA virus, of which there are four serotypes (D1V, D2V, D3V and D4V). The viral genome encodes three structural proteins (C, M and E) and seven non-structural proteins (NS1, NS2a, NS2b, NS3, NS4a, NS4b and NS5). DV is known to cause a spectrum of illnesses, ranging from mild dengue fever (DF) to severe dengue hemorrhagic fever and dengue shock syndrome (DHF/DSS). Currently, DF and DHF/DSS are major global public health problems. It is estimated that 50,000,000–100,000,000 cases of DF and 250,000–500,000 cases of DHF/DSS occur every year worldwide [1].

Despite several decades of research, there are no effective and safe DV vaccines. Previous studies have shown that preexisting DV non-neutralizing antibodies can enhance secondary heterologous DV serotype infections *via* antibody-dependent enhancement (ADE). ADE may be the mechanism for development of DHF/DSS during secondary heterologous DV serotype infections [2-4]. It has been shown that infection with any one DV serotype provides the body with protective immunity against homologous DV serotypes, and with transient cross-protection against heterologous DV serotypes [5]. The majority of studies have demonstrated that interferon gamma (IFN-γ) plays an important role in the clearance of DV following infection [6,7]. Subsequent studies have indicated that DV-specific CD8^+^ T cells display lytic activity and/or produce IFN-γ [8,9]. A recent study in mice confirmed that DV-specific CD8^+^ T cells play a crucial role in controlling DV replication and infection by secreting IFN-γ [10]. Thus, DV-specific CD8^+^ IFN-γ^+^ T cells may be critical for controlling DV infection. However, growing evidence suggests that a DV serotype infection generates not only serotype-specific T cells, but also serotype cross-reactive T cells which can recognize multiple heterologous DV serotypes [9,11-15]. At present, it is accepted that DV serotype-specific T cells provide protective immunity, while serotype cross-reactive T cells induced by primary DV serotype infection are believed to mediate the immunopathogenesis of DHF/DSS during secondary heterologous DV serotype infection [8,16-18].

Because of the important role of serotype-specific CD8^+^ T cells in limiting DV infection, a new strategy for developing prophylactic and therapeutic CD8^+^ T-cell epitope-based vaccines is needed. To avoid the side effect of serotype cross-reactive CD8^+^ T cells, a dengue vaccine must be a tetravalent vaccine that is capable of providing protection against infection by all four DV serotypes simultaneously [19]. Tetravalent CD8^+^ T-cell epitope-based vaccines, which are mixtures of multiple heterologous variant CD8^+^ T-cell epitopes, could be promising candidate vaccines. Although many DV-specific CD8^+^ T-cell epitopes have been identified [9,11,12,17,20-23], the numbers of HLA-A*0201-restricted epitopes are limited, despite the high frequency of the HLA-A*0201 molecule in most populations.

In the present study, we sought to screen the amino acid sequences of D1V and used computational algorithms to predict potential HLA-A*0201-restricted CD8^+^ T-cell epitopes. Candidate CD8^+^ T-cell epitopes and their variant peptides in D2V, D3V, D4V were tested for their affinity to the HLA-A*0201 molecule, and for their capacity to induce CD8^+^ T-cell responses in HLA-A*0201 transgenic mice.

## Results

### Affinity of candidate CD8^+^ T-cell epitopes for HLA-A*0201

**Figure 1:**
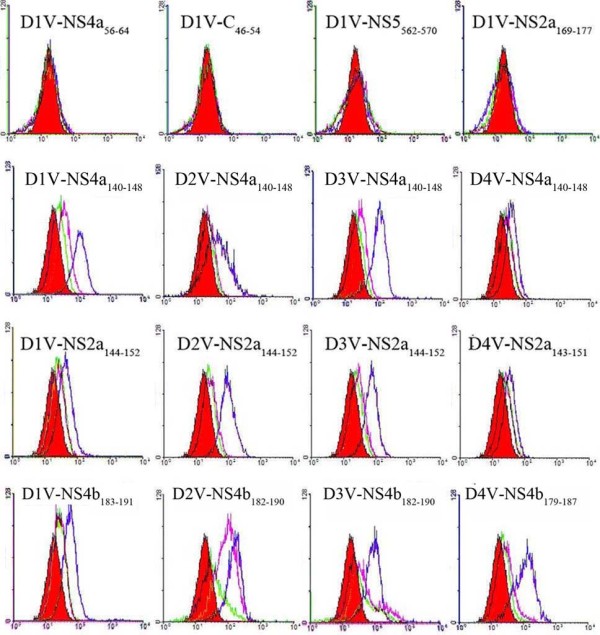
The affinity of candidate epitopes for HLA-A*0201 molecules. T2 cells were incubated in RPMI 1640 medium lacking peptides (*red*) or containing low peptide (1 μg/ml) (*green*) or intermediate peptide (10 μg/ml) (*purple*) or high peptide (100 μg/ml) (*blue*) for 15 h and stained with FITC-conjugated anti-HLA-A*0201 antibody. Then, mean fluorescence intensity (MFI) was measured using flow cytometry.

Seven D1V-derived candidate epitopes [D1V-NS4a_56–64_(MLLALIAVL), D1V-C_46–54_(LVMAFMAFL), D1V-NS4b_562–570_(LLATSIFKL), D1V-NS2a_169–177_(AMVLSIVSL), D1V-NS4a_140-148_(GLLFMILTV), D1V-NS2a_144–152_(QLWAALLSL) and D1V-NS4b_183–191_(LLMRTTWAL)] were synthesized. BLAST results showed that these peptides are highly conserved in more than 100 D1V strains (data not shown). MHC-peptide complex stabilization assay results indicated that three peptides (D1V-NS4a_140-148_, D1V-NS2a_144–152_ and D1V-NS4b_183–191_) showed peptide dose-dependent HLA-A*0201-peptide binding [fluorescence index (FI) exceeds 1 at a peptide concentration of 100 μg/ml], while the other peptides demonstrated weak binding to HLA-A*0201 (FI < 1; Table 1 and Figure 1). According to the sequences for D1V-NS4a_140-148_, D1V-NS2a_144–152_ and D1V-NS4b_183–191_, nine variant peptides derived from D2V, D3V and D4V [D2V-NS4a_140-148_(AILTVVAAT), D3V-NS4a_140-148_(GILTLAAIV), D4V-NS4a_140-148_(TILTIIGLI), D2V-NS2a_144–152_(QLAVTIMAI), D3V-NS2a_144–152_(QLWTALVSL), D4V-NS2a_143–151_(QVGTLALSL), D2V-NS4b_182–190_(LMMRTTWAL)_,_ D3V-NS4b_182–190_(LLMRTSWAL), D4V-NS4b_179–187_(LLMRTTWAF)] were selected for synthesis. BLAST results revealed that each of the nine peptides are highly conserved in more than 80 given serotype strains (data not shown). The variant sequences for D1V-NS4a_140-148_ and D1V-NS2a_144–152_ in D2V, D3V and D4V are highly variable. However, D2V-NS4b_182–190,_ D3V-NS4b_182–190_ and D4V-NS4b_179–187_ all differ from D1V-NS4b_183–191_ by a single amino acid. Regardless of the amino acid variation, almost all nine variant peptides bound to the HLA-A*0201 molecule with high affinity (for D4V-NS2a_143–151_, the FI is 0.8). In total, twelve peptides (D1V-NS4a_140-148_, D2V-NS4a_140-148_, D3V-NS4a_140-148_, D4V-NS4a_140-148_, D1V-NS2a_144–152_, D2V-NS2a_144–152_, D3V-NS2a_144–152_, D4V-NS2a_143–151_, D1V-NS4b_183–191_, D2V-NS4b_182–190_, D3V-NS4b_182–190_ and D4V-NS4b_179–187_) had a high affinity for HLA-A*0201 (Table 1 and Figure 1).

**Table 1:** Candidate epitopes and their affinity for HLA-A*0201 molecule of T2 cells

**Peptides**	**Serotypes**	**Position^a^**	**Sequences^b^**	**Score^c^**	**FI^d^**			**ELISPOT SFCs/1×10^5^cells**	**ICS (%)^e^ CD8^+^IFN-γ^+^T cells**
				**SYFPEITHI**	**1 μg**	**10 μg**	**100 μg**		
D1V-NS4a_56-64_	D1V	NS4a (56-64)	MLLALIAVL	29	0.05	0.05	0.08		
D1V-C_46-54_	D1V	C (46-54)	LVMAFMAFL	20	0.01	0.02	0.02		
D1V-NS4b_562-570_	D1V	NS4b (562-570)	LLATSIFKL	30	0.01	0.05	0.05		
D1V-NS2a_169-177_	D1V	NS2a (169-177)	AMVLSIVSL	28	0.03	0.05	0.04		
D1V-NS4a_140-148_	D1V	NS4a (140-148)	GLLFMILTV	30	0.28	0.85	4.5	58 ± 8	0.71 ± 0.11
D2V-NS4a_140-148_	D2V	NS4a (140-148)	**AI**L**TVV**A**AT**	23	0.1	0.2	1.2	10 ± 3	0.12 ± 0.05
D3V-NS4a_140-148_	D3V	NS4a (140-148)	G**I**L**TL**A**AI**V	24	0.1	0.15	4.05	37 ± 6	0.37 ± 0.08
D4V-NS4a_140-148_	D4V	NS4a (140-148)	**TI**L**TI**I**GLI**	22	0.15	0.3	1.08	17 ± 5	0.21 ± 0.05
D1V-NS2a_144-152_	D1V	NS2a (144-152)	QLWAALLSL	28	0.05	0.22	1.12	36 ± 6	0.41 ± 0.07
D2V-NS2a_144-152_	D2V	NS2a (144-152)	QL**AVTIMAI**	25	0.24	0.39	3.85	21 ± 3	0.21 ± 0.05
D3V-NS2a_144-152_	D3V	NS2a (144-152)	QLW**T**AL**V**SL	29	0.2	0.45	2.6	62 ± 9	0.79 ± 0.15
D4V-NS2a_143-151_	D4V	NS2a (143-151)	Q**VGTLA**LSL	18	0.22	0.2	0.8	12 ± 3	0.13 ± 0.06
D1V-NS4b_183-191_	D1V	NS4b (183-191)	LLMRTTWAL	26	0.29	0.4	1.95	42 ± 7	0.44 ± 0.09
D2V-NS4b_182-190_	D2V	NS4b (182-190)	L**M**MRTTWAL	24	0.58	3.42	4.8	37 ± 5	0.37 ± 0.09
D3V-NS4b_182-190_	D3V	NS4b (182-190)	LLMRT**S**WAL	24	0.22	0.65	3.6	24 ± 5	0.27 ± 0.07
D4V-NS4b_179-187_	D4V	NS4b (179-187)	LLMRTTWA**F**	16	0.2	0.59	4.4	22 ± 4	0.16 ± 0.06

^a^ Position was determined according to the indicated DV serotype.

^b^ Altered peptides representing heterologous DV serotypes. Bolded letters indicate the variant amino acid.

^c^ Score predicted by online prediction algorithms SYFPEITHI.

^d^ FI = [Mean fluorescence intensity(MFI) sample-MFI background]/MFI background.

^e^ The proportion of CD8^+^IFN-γ^+^T cells in CD8^+^Tcells.

### Induction of peptide-specific CD8^+^ T cells in HLA-A*0201 transgenic mice

**Figure 2:**
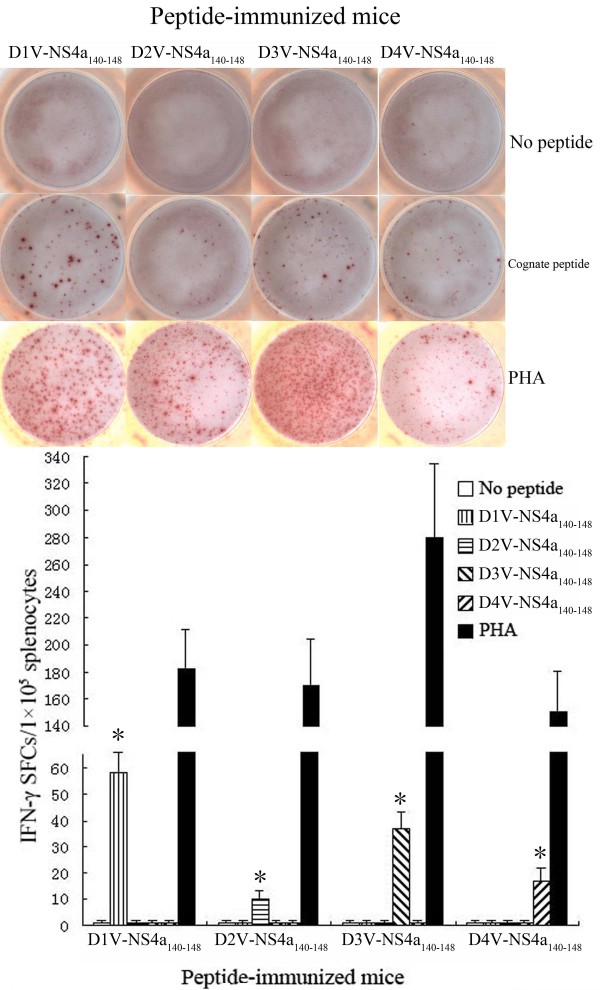
Magnitude of the ELISPOT response to D1V-NS4a_140-148_ and heterologous peptides in HLA-A*0201 transgenic mice immunized with peptides. Splenocytes were isolated from the peptide-immunized mice and were stimulated *in vitro* with cognate peptide or heterologous peptide. The numbers of IFN-γ SFCs/1×10^5^ splenocytes were detected using ELISPOT assay. *Indicating the positive response to a peptide.

**Figure 3:**
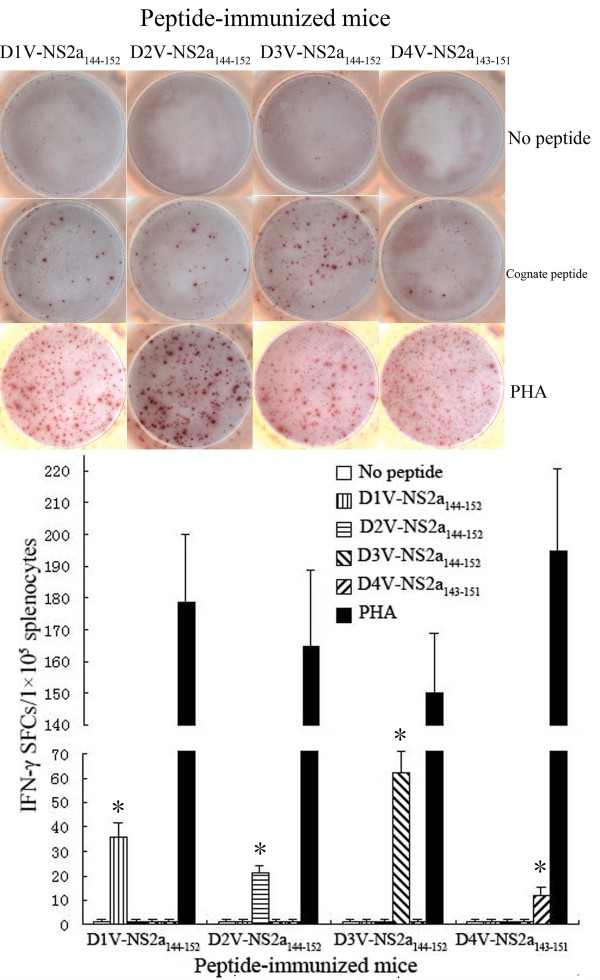
Magnitude of the ELISPOT response to D1V-NS2a_144-152_ and heterologous peptides in HLA-A*0201 transgenic mice immunized with peptides. Splenocytes were isolated from the peptide-immunized mice and were stimulated *in vitro* with cognate peptide or heterologous peptide. The numbers of IFN-γ SFCs/1×10^5^ splenocytes were detected using ELISPOT assay. *Indicating the positive response to a peptide.

**Figure 4:**
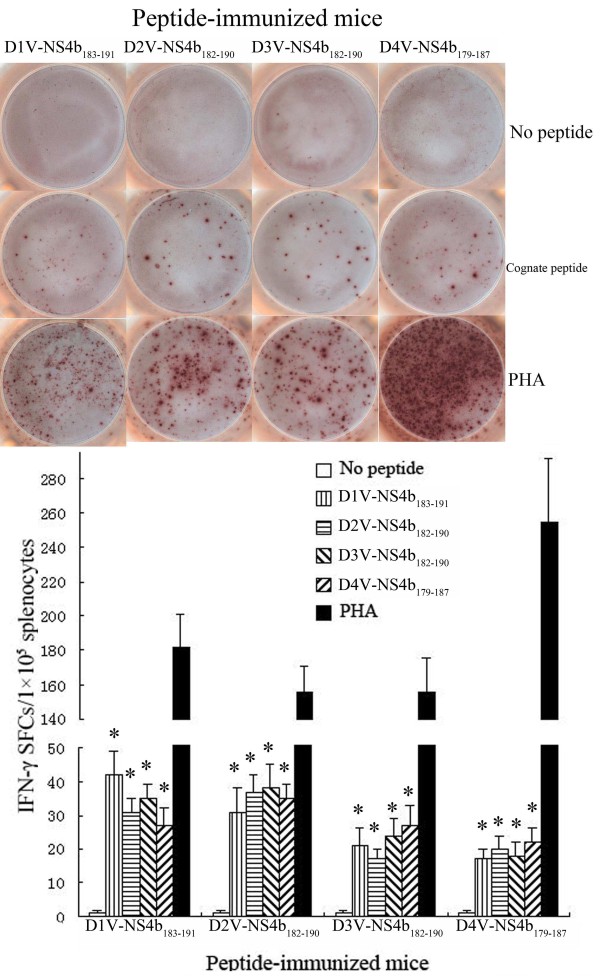
Magnitude of the ELISPOT response to D1V-NS4b_183-191_ and heterologous peptides in HLA-A*0201 transgenic mice immunized with peptides. Splenocytes were isolated from the peptide-immunized mice and were stimulated *in vitro* with cognate peptide or heterologous peptide. The numbers of IFN-γ SFCs/1×10^5^ splenocytes were detected using ELISPOT assay. *Indicating the positive response to a peptide.

**Figure 5:**
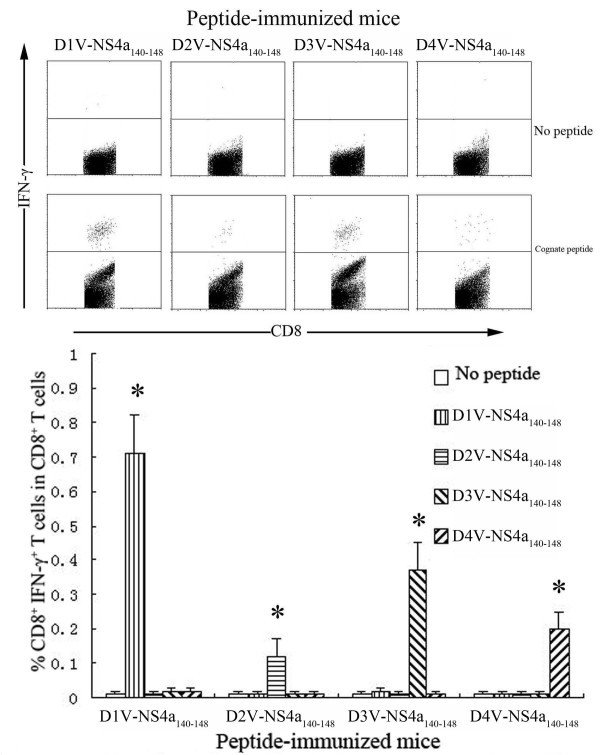
Detection of peptide-specific CD8^+^ IFN-γ^+^ T cells in HLA-A*0201 transgenic mice immunized with D1V-NS4a_140-148_ or heterologous peptides. Splenocytes were isolated from the peptide-immunized mice and were stimulated *in vitro* with cognate peptide or heterologous peptide. The percentages of CD8^+^ IFN-γ^+^ T cells in CD8^+^ T cells were measured using ICS assay. *Indicating the positive response to a peptide.

**Figure 6:**
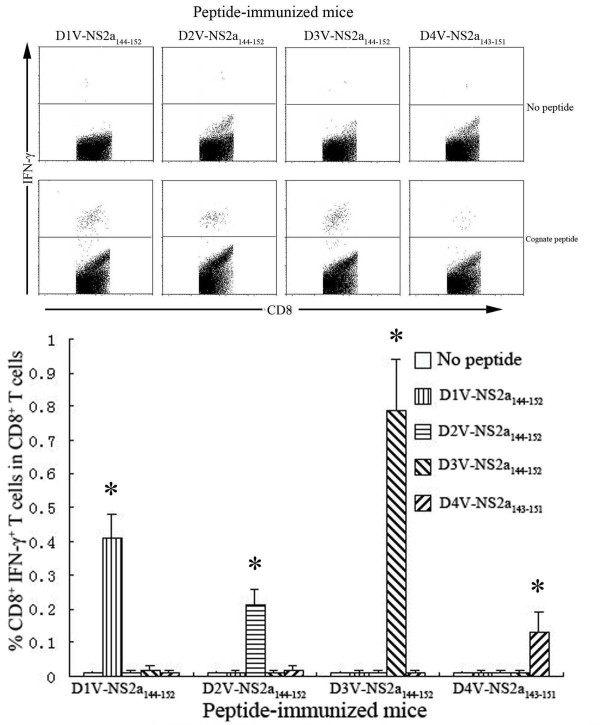
Detection of peptide-specific CD8^+^ IFN-γ^+^ T cells in HLA-A*0201 transgenic mice immunized with D1V-NS2a_144-152_ or heterologous peptides. Splenocytes were isolated from peptide-immunized mice and were stimulated *in vitro* with cognate peptide or heterologous peptide. The percentages of CD8^+^ IFN-γ^+^ T cells in CD8^+^ T cells were measured using ICS assay. *Indicating the positive response to a peptide.

**Figure 7:**
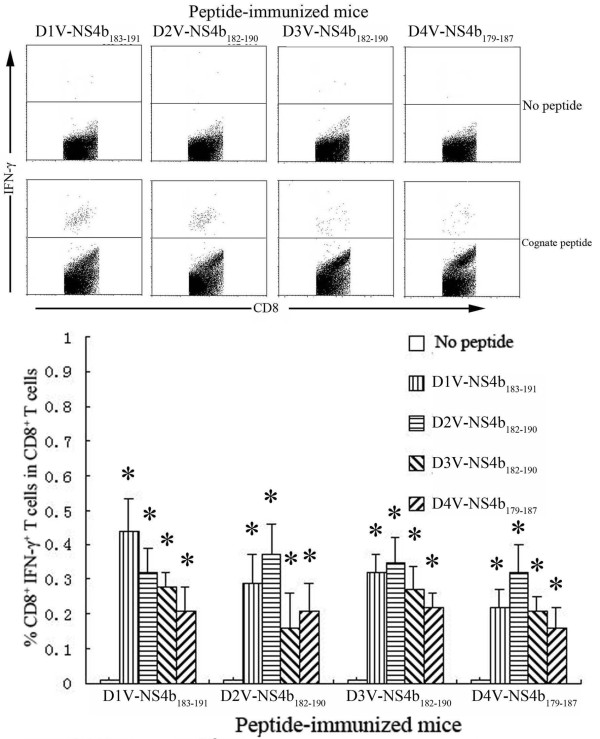
Detection of peptide-specific CD8^+^ IFN-γ^+^ T cells in HLA-A*0201 transgenic mice immunized with D1V-NS2a_183-191_ or heterologous peptides. Splenocytes were isolated from the peptide-immunized mice and were stimulated *in vitro* with cognate peptide or heterologous peptide. The percentages of CD8^+^ IFN-γ^+^ T cells in CD8^+^ T cells were measured using ICS assay. *Indicating the positive response to a peptide.

To evaluate the immunogenicity of high-affinity peptides, splenocytes isolated from HLA-A*0201 transgenic mice immunized with peptide were stimulated with a cognate peptide. Enzyme-linked immunospot (ELISPOT) and intracellular cytokine staining (ICS) assays were used to detect homologous peptide-specific CD8^+^ T cells. ELISPOT results revealed that immunized HLA-A*0201 transgenic mice had high levels of peptide-specific IFN-γ spot-forming cells (SFCs) in splenocytes. All peptides evoked an IFN-γ response in the ELISPOT assay that exceeded 10 SFCs/1 × 10^5^ splenocytes. D3V-NS2a_144–152_ immunization resulted in the highest frequency of IFN-γ-secreting cells (62 ± 9 SFCs/1 × 10^5^ splenocytes), while D2V-NS4a_140-148_ immunization showed the lowest frequency of IFN-γ-secreting cells (10 ± 3 SFCs/1 × 10^5^ splenocytes). As for D1V-NS4a_140-148_ and its variant peptides, with the increase of FI, higher level of peptide-specific IFN-γ-secreting cells was induced. In contrast, for D2V-NS2a_144-152_, D1V-NS4b_183-191_ and their variant peptides, the frequencies of peptide-specific IFN-γ-secreting cells were not proportional to peptides’ FIs. For example, the FIs of D1V-NS4a_140-148_ and D3V-NS4a_140-148_ were 4.5 and 4.05, respectively. These two peptides all induced higher level of IFN-γ-secreting cells (58 ± 8 SFCs and 37 ± 6 SFCs, respectively). Although D2V-NS2a_144-152_ and D4V-NS4b_179-187_ had higher FIs, they just induced median level of IFN-γ-secreting cells (21±3 SFCs and 22±4 SFCs, respectively). A summary of the individual peptides that evoked responses in IFN-γ ELISPOT assays is provided in Table 1 and Figures 2, 3, and 4. The ICS results defined the phenotype of peptide-specific IFN-γ-secreting T cells, and confirmed the human leukocyte antigen (HLA) restriction of these peptides. The high frequencies of CD8^+^ IFN-γ^+^ T cells were detected in splenocytes after being stimulated with cognate peptide. The highest percentage of CD8^+^ IFN-γ^+^ T cells was specific for D3V-NS2a_144–152_ (0.79 ± 0.15%). The lowest percentage of CD8^+^ IFN-γ^+^ T cells was directed against D2V-NS4a_140-148_ (0.12 ± 0.05%; Table 1, Figures 5, 6, and 7). The ELISPOT results were consistent with the ICS results. Additionally, these peptides were tested in an IFN-γ ELISPOT assay and an ICS assay using splenocytes from mock-immunized HLA-A*0201 transgenic mice. They were also tested using splenocytes from peptide-immunized C57BL/6 mice. Significant responses were not observed in either of these situations (data not shown).

### Cross-reactivity of peptide-specific CD8^+^ T cells

To further explore the cross-reactivity between a given peptide and its variants, we examined the ability of peptide-specific CD8^+^ T cells to recognize a heterologous peptide variant representing another DV serotype. Splenocytes from D1V-NS4b_183–191_-immunized mice exhibited marked cross-reactivity towards D2V-NS4b_182–190_, D3V-NS4b_182–190_ and D4V-NS4b_179–187_. Similar data were obtained for D2V-NS4b_182–190_, D3V-NS4b_182–190_ and D4V-NS4b_179–187_ (Figures 4 and 7). The proportion of CD8^+^ IFN-γ^+^ T cells responding to all four peptides ranged from 0.16–0.44%. For D1V-NS4b_183–191_ and D2V-NS4b_182–190_, the same variant peptides induced the highest CD8^+^ T-cell response in all peptide-immunized mice. In total, higher responses to homologous peptides were more common than responses to variant peptides. For the remaining eight peptides, stimulation of splenocytes with their corresponding variants did not give rise to CD8^+^ IFN-γ^+^ T cells.

## Discussion

Both our previous reports and other studies indicate that combining prediction algorithms with several *in vitro* and/or *in vivo* assays could hasten the identification of immunogenic T-cell epitopes [21,24]. It is established that HLA-A*0201 is the major haplotype in most of the world population, irrespective of gender and race [25]. Therefore, HLA-A*0201-restricted CD8^+^ T-cell epitopes would likely have broad population coverage.

In the present study, seven D1V-derived potential HLA-A*0201-restricted candidate epitopes were evaluated for their binding capacity to HLA-A*0201. Three peptides were identified as high-affinity peptides. Almost all variants of these three peptides in D2V, D3V, D4V have a high affinity for HLA-A*0201. In total, twelve peptides demonstrated a high affinity for HLA-A*0201.

Classic HLA-A*0201-restricted epitopes have an L or I amino acid residue at position 2, and an L, I or V residue at position 9. Among sixteen candidate epitopes described here, D1V-NS4a_140-148_, D3V-NS4a_140-148_, D4V-NS4a_140-148_, D1V-NS2a_144–152_, D2V-NS2a_144–152_, D3V-NS2a_144–152_, D1V-NS4b_183–191_ and D3V-NS4b_182–190_ followed this classic pattern. They also showed a high affinity for HLA-A*0201 (FI > 1). D1V-NS4a_56–64_ and D1V-NS4b_562–570_ had a low-affinity for HLA-A*0201, even though they shared these classic residues at the relevant positions (FI < 0.5). In contrast, although neither D2V-NS4b_182–190_ (position 2 is M) nor D4V-NS4b_179–187_ (position 9 is F) conformed to the classic pattern, these peptides had a high affinity for HLA-A*0201 (FI > 4). A possible explanation for these phenomena may be that amino acids in other positions drastically affect binding avidity.

To further evaluate the immunogenicity and HLA allele restriction of these high-affinity peptides, we assessed whether these twelve peptides could elicit CD8^+^ T-cell responses in HLA-A*0201 transgenic mice. Although these twelve peptides had different affinities for HLA-A*0201, they all triggered peptide-specific CD8^+^ T cell responses. The magnitude of responses to individual peptides ranged from 10–62 SFCs/1×10^5^ splenocytes. Our results appear to correspond with those seen in other studies. The frequencies of CD8^+^ IFN-γ^+^ T cells that respond to cognate peptides in splenocytes of HLA-A*0201 transgenic mice (0.12–0.79% CD8^+^ IFN-γ^+^ T cells of all CD3^+^ CD8^+^ T cells) is in line with frequencies detected in humanized mice (0.1–2.8% of all CD3^+^ CD8^+^ T cells) [26,27], and in human peripheral blood mononuclear cells (PBMCs) from DV immune donors (0.1–0.68% of all CD3^+^ CD8^+^ T cells) [12,21]. These peptides did not induce significant CD8^+^ T-cell responses in mock-immunized HLA-A*0201 transgenic or C57BL/6 mice (data not shown). These data further confirmed that these twelve peptides were recognized by HLA-A*0201.

Additionally, a high frequency of D1V-NS4a_140-148_ and D3V-NS2a_144–152_-specific CD8^+^ T cells in peptide-immunized HLA-A*0201 transgenic mice suggests that these peptides might be the immunodominant HLA-A*0201-restricted epitopes. In recent years, research studies have revealed many DV-specific CD8^+^ T-cell epitopes. These are mostly located in E, NS3, NS4a, NS4b, NS5 and restricted by HLA-A2, A11, A24, B7, B55, B65 [9,11,12,17,20-23]. Lund et al [22] and Weiskopf et al [23] used HLA-A*0201 transgenic mice and identified several D1V- and D2V-specific HLA-A*0201-retricted epitopes, respectively. Weiskopf et al [23] also found that most of the epitopes identified in the murine system are also recognized by PBMCs from DV-exposed human donors. In the present study, based on a similar strategy, we identified NS2a-, NS4a- and NS4b-derived HLA-A*0201-restricted CD8^+^ T-cell epitopes from D1V–D4V. Our results suggest that NS2a, NS4a and NS4b are involved in cellular immunity during DV infection.

Previous studies have shown that DV-specific CD8^+^ T cells from DV-immunized or infected subjects exhibited a cross-reactive response to variant peptides representing a heterologous serotype [9,12,15]. In this study, for D1V-NS4b_183–191_ and its variant peptides (D2V-NS4b_182–190_, D3V-NS4b_182–190_ and D4V-NS4b_179–187_) each elicited peptide-specific CD8^+^ T cells, which exhibited cross-reactivity towards its variants. These data suggest that D1V infection followed by D2V, D3V or D4V infection (or vice versa) would trigger the activation of cross-reactive IFN-γ-producing CD8^+^ T cells. Cross-reactivity may be explained by the primary structure of these variants: D2V-NS4b_182–190_(LMMRTTWAL), D3V-NS4b_182–190_(LLMRTSWAL), and D4V-NS4b_179–187_(LLMRTTWAF). Each of these differs from D1V-NS4b_183–191_(LLMRTTWAL) by a single amino acid. D1V-NS4b_183–191_ and D3V-NS4b_182–190_ shared the same anchors at positions 2 (L) and 9 (L). These are critical positions for HLA recognition and T-cell activation. These two peptides have only one amino acid change at position 6 (T→S). For D2V-NS4b_182–190_ and D4V-NS4b_179–187_, the residues at positions 2 or 9 differ from D1V-NS4b_183–191_ and D3V-NS4b_182–190_. We believe that the amino acid change will not affect functional avidity (IFN-γ secretion). In a recent study using HLA-A*0201-positive D1V/D2V/D3V-immune donors, Bashyam et al [12] reported four cross-reactive epitopes (D1V-ILLMRTTWA, D2V-VLLMRTTWA, D3V-LLLMRTSWA and D4V-LLLMRTTWA). In comparison, we found those epitopes along with the ones reported in this paper to share 7 or 8 amino acids. We are confident that the shared amino acid sequences may determine HLA-A*0201 restriction and T cell recognition.

## Conclusions

In summary, based on the amino acid sequences of D1V-D4V, we identified two novel serotype-specific HLA-A*0201-restricted CD8^+^ T-cell epitopes (NS4a_140-148_ and NS2a_144–152_) and one cross-reactive HLA-A*0201-restricted CD8^+^ T-cell epitopes which is similar to a previously identified epitope. In the following study, we would explore whether these peptide could be recognized by PBMCs from human donors infected with DV. Our results show that using a combination of prediction algorithms and HLA transgenic mice is effective for identifying HLA-restricted epitopes. In general, the antiviral activity of CD8^+^ T cells is mediated by the production of cytokines, particularly IFN-γ. Further studies will be needed to determine the protective role of these serotype-specific epitopes. D1V-NS4b_183–191_, D2V-NS4b_182–190_, D3V-NS4b_182–190_ and D4V-NS4b_179–187_ cross-reacted with each other, therefore further evaluation of the functional phenotype of serotype cross-reactive CD8^+^ T cells induced by these peptides would reveal the exact mechanism of T cell-mediated immunopathogenesis during secondary heterologous DV serotype infection.

## Methods

### Epitope prediction and peptide synthesis

Based on the amino acid sequence of D1V (Hawaii strain; GenBank Accession No: ACF49259), the epitope prediction algorithms SYFPEITHI with PAProc (http://www.syfpeithi.de; http://www.paproc.de) were applied to predict HLA-A*0201-restricted CD8^+^ T-cell epitopes. The following criteria were used to select candidate CD8^+^ T-cell epitopes. First, the candidate epitope should be a nonapeptide that has a high predictive score and a protease cleavage site (C terminus). Second, the sequence of the candidate epitope should be highly conserved in most D1V strains. If a candidate epitope has a high affinity for HLA-A*0201 as confirmed by an MHC peptide complex stabilization assay, its variant peptides in D2V (NGC strain; AAC59275), D3V (H87 strain; AAA99437) and D4V (H241 strain; AAX48017) would be selected and synthesized. All peptides were synthesized at > 90% purity by ChinaPeptides Co., Ltd (Shanghai, China).

### Cells and mice

The transporter associated with antigen processing (TAP)-deficient T2 cell line was purchased from ATCC (Manassas, VA, USA). Female C57BL/6-Transgenic(HLA-A2.1)1Enge/J mice (HLA-A*0201 transgenic mice; 6–8 weeks) were purchased from the Jackson Laboratory (Bar Harbor, ME, USA). Female C57BL/6 mice (6–8 weeks) were provided by the Laboratory Animal Center of Wenzhou Medical College.

### MHC-peptide complex stabilization assay

The ability of peptides to stabilize MHC molecules on the surface of T2 cells was measured as described previously [21]. T2 cells (2 × 10^5^ cells/0.5 ml) were incubated in serum-free RPMI 1640 medium in the presence of different concentrations of peptide (1, 10, 100 μg/ml) for 1 h at 37°C/5% CO_2_ incubator. Cells were then incubated at 26°C for 12 h, and then returned to 37°C for a 3 h incubation. Finally, T2 cells were stained with FITC-conjugated anti-HLA-A*0201 antibody (BD Pharmingen, USA) for 40 min at 4°C. T2 cells incubated in serum-free RPMI 1640 medium without peptides served as a negative control (background). Mean fluorescence intensity (MFI) of T2 cells was recorded with a FACS Calibur flow cytometer (BD bioscience, USA), and results expressed as fluorescence index (FI). The following formula was used to calculate the FI.

(1) $$\text{FI}=\frac{\text{MFI sample}-\text{MFI background}}{\text{MFI background}}$$

### Immunization of HLA-A*0201 transgenic mice

HLA-A*0201 transgenic mice were subdivided into 12 groups (4 mice/group). Mice were inoculated subcutaneously with high-affinity peptide (50 μg/mouse) emulsified in Freund’s complete adjuvant. One week later, mice were immunized with the same peptide emulsified in Freund’s incomplete adjuvant. Mice were boosted three more times at weekly intervals. Mock-immunized (adjuvant alone) HLA-A*0201 transgenic mice and peptide-immunized C57BL/6 mice served as controls. One week after the final immunization, all mice were sacrificed and splenocytes extracted. ELISPOT and ICS assays were conducted to detect the frequencies of peptide-specific IFN-γ-producing cells. All animal were performed following the Institutional Animal Care and Use Committee-approved protocols.

### IFN-γ ELISPOT assays

Splenocytes were resuspended to a final concentration of 1 × 10^6^ cells/ml in RPMI 1640 medium supplemented with 10% fetal bovine serum (FBS). ELISPOT assays were performed in pre-coated 96-well plates (U-CyTech Company, Netherlands). Splenocytes were seeded at 1 × 10^5^ cells/well and exposed to either cognate or heterologous peptide at a final concentration of 10 μg/ml. Negative control wells contained splenocytes but no peptide. Positive control wells included cells plus phytohemagglutinin (PHA) at a final concentration of 10 μg/ml. All tests were carried out in duplicate wells, with plates incubated for 24 h at 37°C/5% CO_2_. Plates were washed and then incubated with biotinylated anti-mouse IFN-γ for 1 h at 37°C. After washing, plates were labeled with streptavidin-horseradish peroxidase, and developed using fresh ACE solution as a substrate. IFN-γ spots were counted using an ELISPOT reader (Beijing SageCreation Science Co. Ltd, Beijing, China). Peptide-specific T-cell frequency was expressed as SFCs/1 × 10^5^ splenocytes. Background spots (negative control wells) were subtracted from test wells. A positive response to a peptide was defined as having > 5 SFCs/1 × 10^5^ splenocytes after subtraction of the background.

### ICS assays

Splenocytes were cultured with either cognate peptide (10 μg/ml) or heterologous peptide (10 μg/ml) in a 1.5 ml microcentrifuge tube for 6 h at 37°C/5% CO_2_. Negative controls did not receive any peptide stimulation. During the last 5 h, brefeldin A (10 μg/ml) was added to each tube. After a 6 h incubation, cells were washed and then stained with APC-conjugated anti-mouse CD3 and FITC-conjugated anti-mouse CD8 antibodies (eBioscience company, USA) for 40 min at 4°C. Cells were then washed and fixed with 4% paraformaldehyde for 20 min at 4°C, permeabilized using 0.5% saponin for 10 min at 4ºC, and stained with PE-conjugated anti-mouse IFN-γ antibody (eBioscience company, USA) for 40 min at 4°C. A FACS Calibur flow cytometer (BD Bioscience, USA) was used to analyze labeled cells. CD3^+^ CD8^+^ T cells were gated and the proportion of IFN-γ-producing CD8^+^ T cells (CD8^+^ IFN-γ^+^ T cells) as a subset of all CD8^+^ T cells were determined.

### Statistical analysis

Data are expressed as mean value ± standard deviation (SD). The Student’s t-test was used to test statistical significance. P values of < 0.05 were considered statistically significant.

## Competing interests

The authors declare that they have no competing interests.

## Authors' contributions

ZLD, QL and JSW conceived of the study. ZLD performed MHC-peptide complex stabilization assay and murine IFN-γ ELISPOT assay, analyzed the results and drafted the manuscript; QL performed mice vaccination and ICS assay. JSW supervised the research and helped draft the manuscript. ZBW and KDX assisted with data analysis. JLG and WQL prepared the experiment. Manuscript is approved by all authors for publication.
